# Implementation of an Electronic Medication Management System in 41 Residential Care Homes in Hong Kong: Pre–Post Interventional Study

**DOI:** 10.2196/79262

**Published:** 2025-12-04

**Authors:** Phillip Lung Wai Au-Doung, Ho Cheung Chau, Chit Hin Wong, Kai Cheong Lau, Yi Qi Chan, Ho Lam Yip, Chiu Ngai Hung, Bik-Wai Bilvick Tai, Teddy Tai-Ning Lam, Chui Ping Lee, Sau Chu Chiang, Yin Ting Cheung

**Affiliations:** 1 School of Pharmacy, Faculty of Medicine, The Chinese University of Hong Kong, Lo Kwee-Seong Integrated Biomedical Sciences Building, Hong Kong, China (Hong Kong), 852 39346833; 2 Hong Kong Pharmaceutical Care Foundation Limited, Hong Kong, China (Hong Kong)

**Keywords:** medication management, residential care homes, electronic medication records, time motion study, cost saving, older adults, nursing homes

## Abstract

**Background:**

The medication management process in resident care homes for the elderly (RCHEs) is complex and can be labor intensive. In 2019, a nongovernment organization led by pharmacists with special interest in informatics developed the SafeMed Medication Management System (SMMS), which is a digital web-based system that integrates electronic medical profiles and medication profiles to revamp the traditional manual medication management process in RCHEs in Hong Kong.

**Objective:**

This study aimed to assess the effectiveness of the SMMS in improving RCHE staff’s time efficiency and competencies in the medication management process and how this could potentially reduce human resource costs after its implementation.

**Methods:**

This was a pre–post interventional study conducted from September 2022 to August 2024. Time efficiency was evaluated using time–motion analysis. The time spent on each process—preparing the medication doses, checking the prepared doses, and administering the doses to residents—was evaluated in 10-minute blocks. The mean numbers of doses prepared, checked, and administered were calculated for each block. A three-way ANOVA was used to compare the doses before and after the system implementation. Staff competencies and perceived acceptance of the system were evaluated using a structured survey adapted from the technology acceptance model. An exploratory analysis was conducted to estimate the potential financial savings attributable to SMMS implementation using RCHE staff salary data obtained from publicly available governmental sources. Results from the time–motion analysis were used to estimate the cost per dose prepared by RCHE staff before and after the system implementation.

**Results:**

Forty-one RCHEs implemented the SMMS, serving a total of 3911 residents. The time–motion analysis (n=6 RCHEs) revealed that the mean (SD) number of doses significantly increased in 10-minute blocks after system implementation (medication preparation: 25, SD 14 to 49, SD 15 doses; medication checking: 21, SD 6 to 85, SD 33 doses; medication administration: 9, SD 1 to 16, SD 6 doses). The overall mean number of doses handled across all processes combined was significantly higher after implementation (18.9 vs 51.9 doses, *P*=.02). RCHE staff (n=392) reported significantly improved competencies in entering and accessing residents’ records and preparing, checking, and administering medications after the system implementation (all *P*<.001). The estimated cost of managing one dose of medication dropped substantially from HKD 2.00 (US $0.25) before to HKD 0.74 (US $0.09) after system implementation. If fully implemented in all RCHEs across Hong Kong, the daily human resource cost associated with the medication management process could potentially be reduced from HKD 2,574,000 (US $330,000) to HKD 952,380 (US $122,100).

**Conclusions:**

The time–motion analysis and quantitative survey findings suggest that digital technology combined with automation can improve staff’s time efficiency and competencies and promote human resource cost-saving in the medication management process. Future work should evaluate the long-term impact of this system on medication safety and its cost-effectiveness in RCHEs.

## Introduction

Hong Kong has a rapidly aging population. From 1997 to 2023, the number of people aged 65 years and above more than doubled to 1.68 million, representing 22.4% of the total population [1]. Currently, there are around 74,900 residential care places across the city, with occupancy of approximately 58,500 residents [2]. In addition to adopting a multipronged approach to increase the number of resident care homes for the elderly (RCHEs), improving the standard of health care in RCHEs has become a top priority in Hong Kong.

The working environment in RCHEs is often busy and characterized by a heavy workload [3,4]. The dynamic nature of the workplace necessitates that staff prioritize tasks and perform multiple activities simultaneously while performing medication management [3,4]. In Hong Kong, medication management in RCHEs is mostly handled manually, making the process highly inefficient and labor-intensive. The RCHE residents receive regular medical follow-ups from the public hospitals and clinics under the Hong Kong Hospital Authority (HA). However, the electronic prescription records of HA are not synced with or integrated into the medication administration records in RCHEs. Therefore, the medication management tasks in RCHEs often include manually transcribing, preparing, dispensing, and checking medications, as well as managing documentation on medication administration records [5,6]. According to the literature, various medication errors occur in RCHEs, including errors in preparing drugs, their quantities, and the timing of dispensing medications [7-10]. Such errors could cause serious medication-related problems to RCHE residents, who tend to be vulnerable because of functional decline, multimorbidity, and polypharmacy [11-13]. There is therefore a crucial need to improve the efficiency of medication management without compromising patient safety.

Human-related medication errors can be minimized by incorporating IT [14]. For example, the use of electronic medical profiles and electronic medication records has been shown to reduce errors [15,16] by identifying mismatches in medication records and alerting RCHE staff automatically [17]. The use of electronic medication administration records (eMARs) is associated with a significantly lower rate of medication administration errors than the use of paper-based records [16]. Furthermore, the use of technology can potentially improve the efficiency of the medication management workflow as administrative paperwork is replaced electronically [17,18]. Digitalization allows instant access to residents’ medication profiles [15], enabling the immediate tracing and checking of records [18]. By leveraging the power of IT, RCHE staff can enhance the safety and efficiency of workflows, thereby minimizing the risk of medication errors and improving patient outcomes.

In 2019, a group of pharmacists specializing in clinical informatics was appointed by a local nongovernmental organization, the Hong Kong Pharmaceutical Care Foundation Ltd (HKPCF), to develop the SafeMed Medication Management system (SMMS) [19]. The SMMS is a digital system that integrates electronic medical profiles and eMARs. A pilot study showed that the SMMS improved time efficiency and reduced medication errors [5]. However, the pilot study only included two RCHEs and did not evaluate the system from the perspectives of RCHE staff. Hence, the findings of the pilot study might not be generalizable to other RCHEs, and the study lacked a crucial perspective on the system’s usability and effectiveness from the standpoint of RCHE staff who would be using it daily.

The primary objectives of this study were to evaluate the system’s effects in two key areas: (1) to assess the effectiveness of the SMMS in improving time efficiency in the medication management process and (2) to examine the RCHE staff’s competencies and perceived acceptance of the SMMS. To provide a financial perspective on medication management, an exploratory analysis was also conducted to estimate the potential cost savings in human resources after the implementation of the SMMS.

## Methods

### Study Design

This was a pre–post interventional study conducted in the participating RCHEs from January 2022 to August 2024. An invitation was extended to all RCHEs across various geographical regions of Hong Kong to participate in the Integrated Old Age Home Medication Management Program initiated by the HKPCF. The eligible RCHEs had to meet the SMMS criteria, that is, they (1) had a Wi-Fi network; (2) were receiving continued operational funding from the government, the private sector, or philanthropic organizations; and (3) consented in writing to provide deidentified data for evaluation and research purposes. All participating RCHEs were included in the RCHE staff survey, with a subset being further recruited for the time–motion analysis.

### Ethical Considerations

The study was performed in accordance with the Declaration of Helsinki and was approved by the Survey and Behavioral Research Ethics Committee of the Chinese University of Hong Kong (reference 033‐22). The ethics board agreed to waive written informed consent from each RCHE resident individually as one of the terms and conditions in the service agreement. RCHEs receiving the service had agreed to the collection and use of anonymized medication records available in SMMS, prior to the implementation of the Integrated Old Age Home Medication Management Programme. Verbal consent (Table S1 in Multimedia Appendix 1) was further obtained from the RCHE’s superintendents and participating RCHE staff prior to participating in the time–motion analysis. HKPCF pharmacists provided the RCHE staff with regular training and technical assistance in using the SMMS. No compensation was given to the participants.

### Intervention

The SMMS is a digital web-based system that integrates electronic medical profiles and eMARs. In Hong Kong, RCHE residents primarily receive follow-up care from clinicians at public hospitals or general outpatient clinics (GOPCs). These hospitals and GOPCs are managed by the HA, providing over 80% of the medical services to the older people and those with chronic illnesses in Hong Kong. To a lesser extent, general practitioners and clinicians from private clinics or hospitals may also prescribe medications for patients. The pharmacies in HA or private institutions dispense the medications to the patients. Notably, the electronic medical and medication profiles in both the public health care system and private institutions are currently not integrated with the medication management system in the RCHEs.

**Figure 1.**
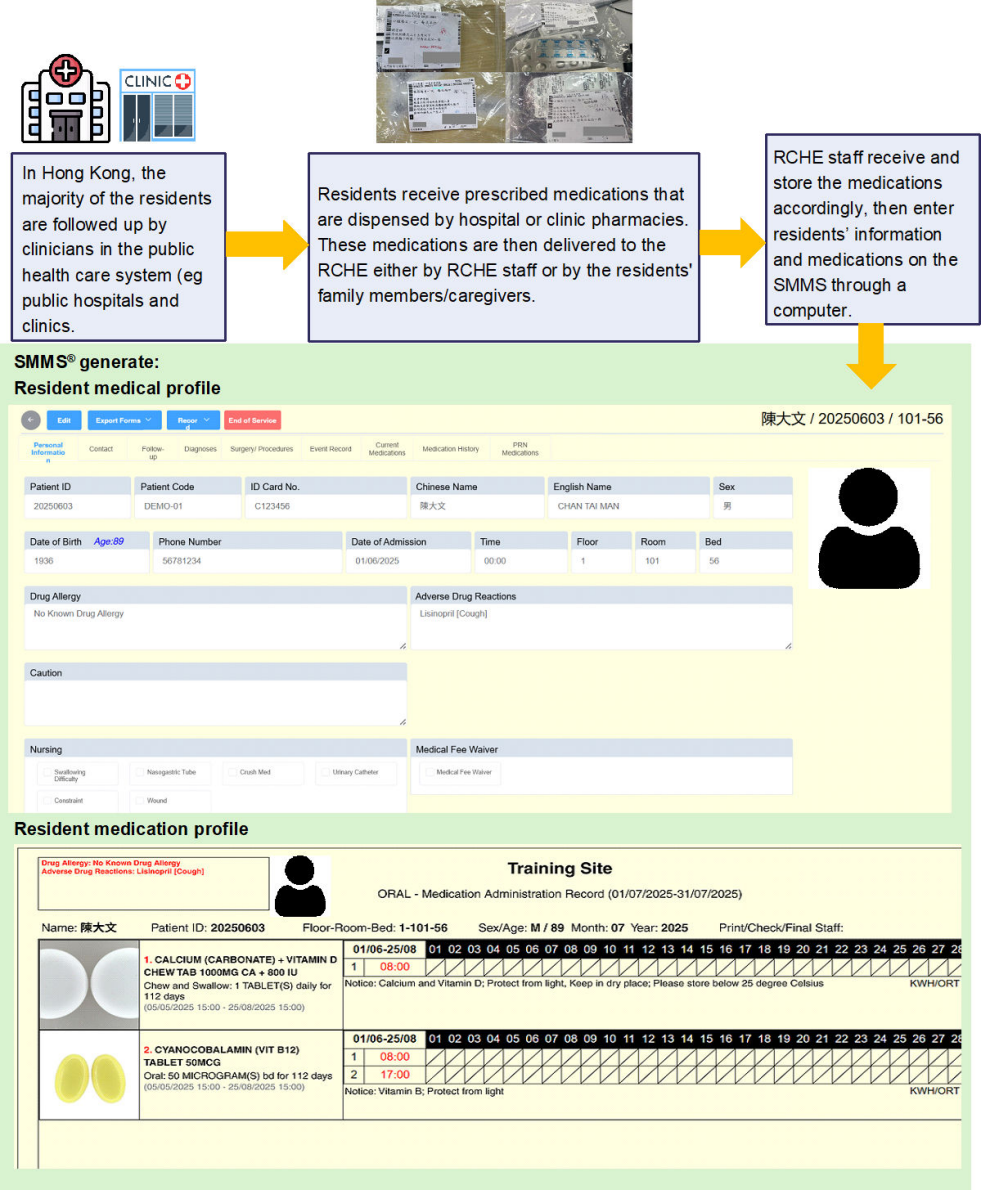
Use of the SMMS in the medication management workflow: generating a medical profile and medication profile of resident, After receiving the medications, RCHE staff enter the information into SMMS. It is able to generate a medical and medication profile for residents with a standardized format. The medication profile is supported by a drug database with drug images by Hong Kong registration number. The database is maintained by pharmacists and covers the majority of medications from the Hong Kong Hospital Authority. RCHE: resident care homes for the elderly; SMMS: SafeMed Medication Management System.

**Figure 2.**
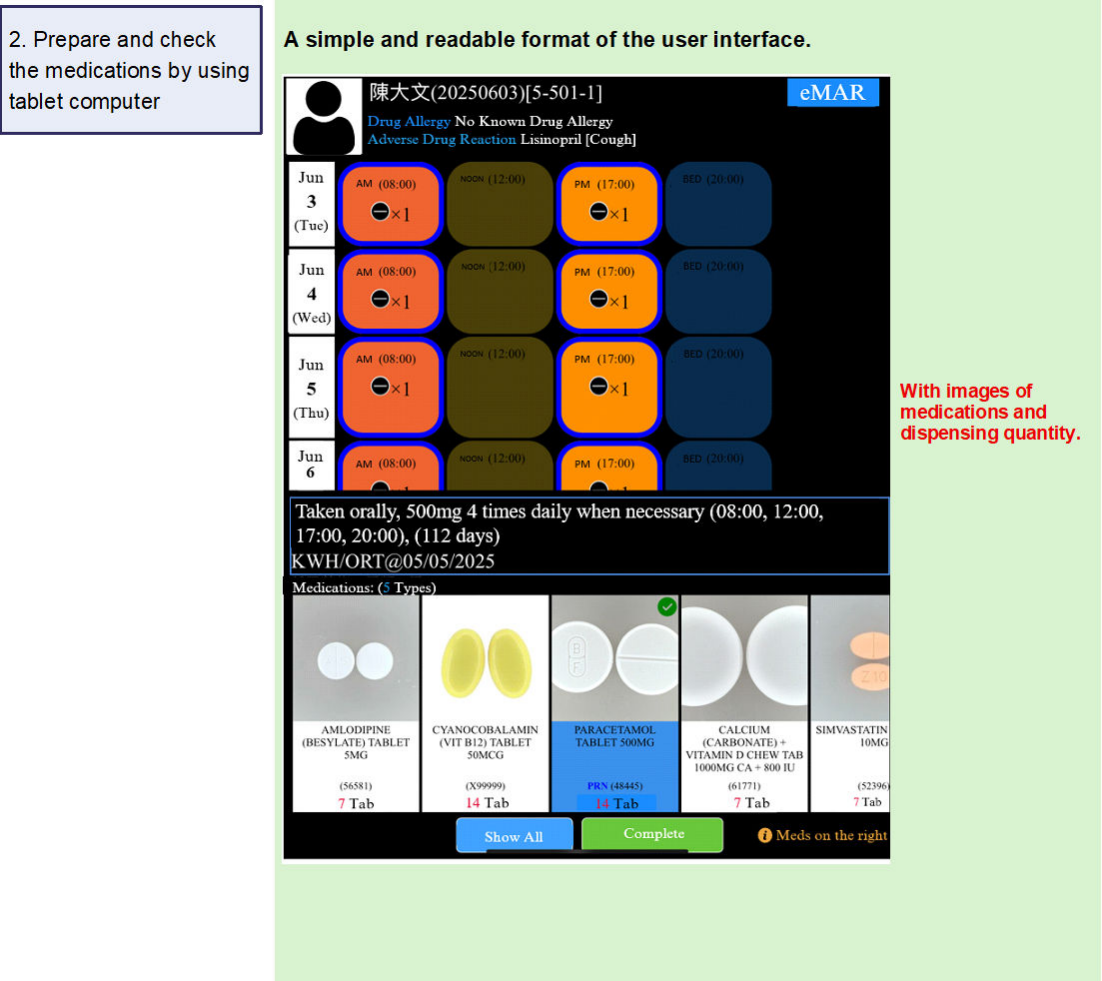
Use of the SMMS in the medication management workflow: conducting medication preparation and checking. RCHE staff prepare and check the medications by using a tablet computer. They can verify the medications by comparing them with the images provided by the system. An electronic signature will be generated automatically after clicking “complete.” eMAR: electronic medication administration record; SMMS: SafeMed Medication Management System.

**Figure 3.**
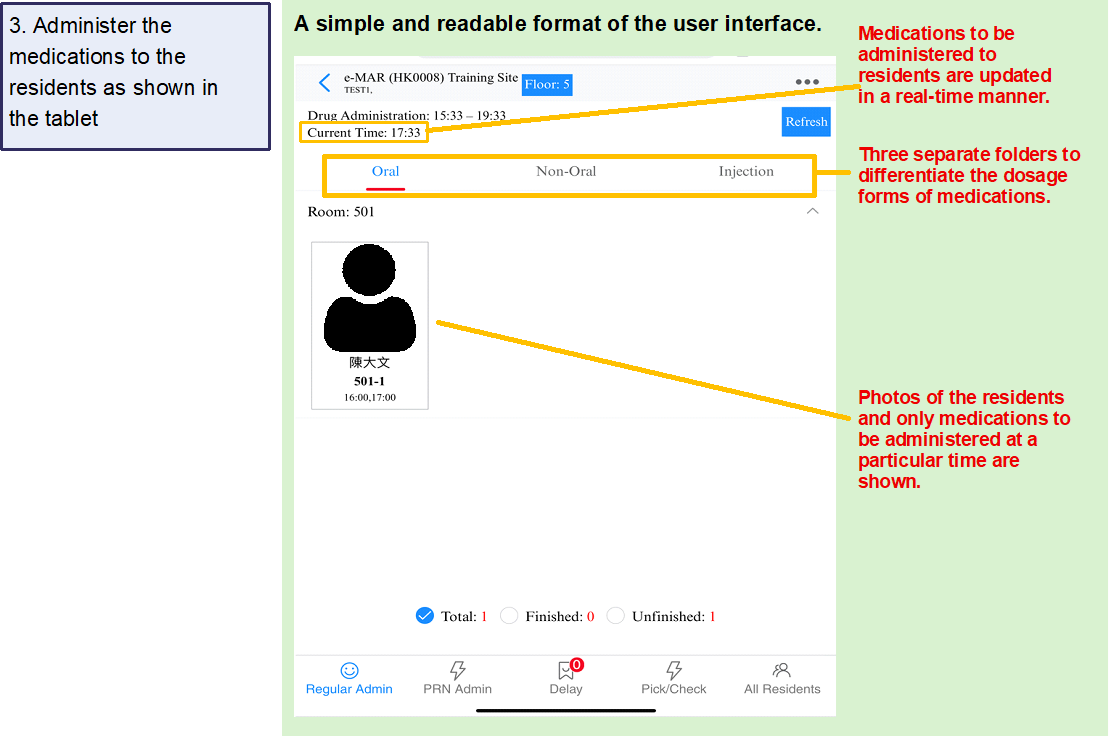
Use of the SMMS in the medication management workflow: conducting medication administration. RCHE staff use the tablet to verify the residents’ details and medications before administering them to the residents. There are three separate folders (oral, nonoral, and injection) to differentiate the dosage forms of medications. RCHE staff can focus on each of the three routes, as each route may require different grades of RCHE staff to execute or supervise the administration. Photos of the resident and medications to be administered at that timeslot will be shown. eMAR: electronic medication administration record; RCHE: resident care homes for the elderly; SMMS: SafeMed Medication Management System.

**Figure 4.**
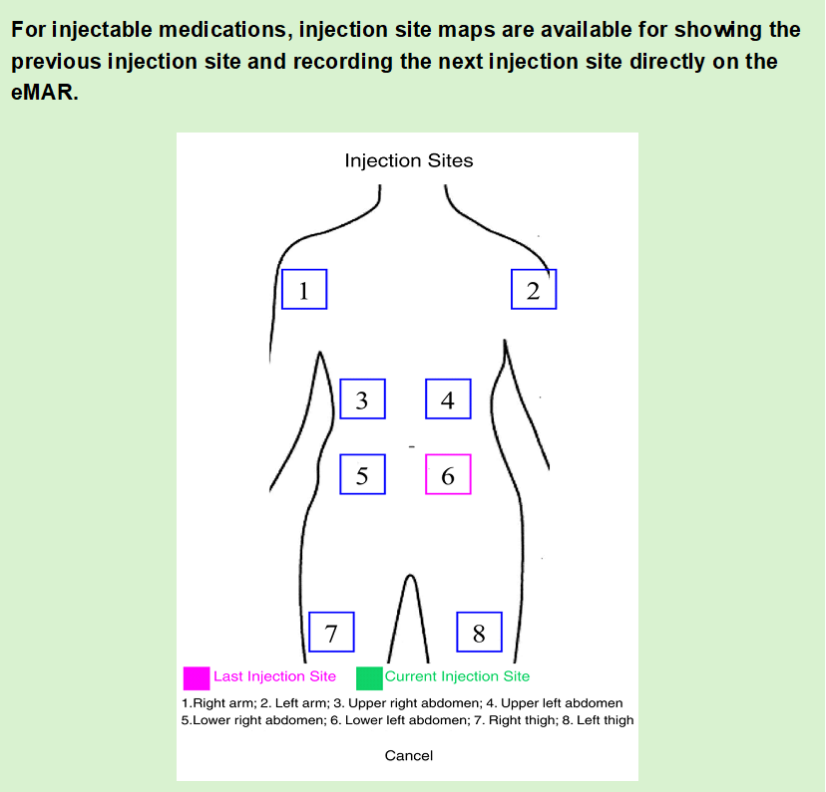
Use of the SMMS in the medication management workflow: conducting medication administration of injectable medications. When administering injectable medications, RCHE staff record the injection site from the tablet to select the appropriate site for the next injection. eMAR: electronic medication administration record; RCHE: resident care homes for the elderly; SMMS: SafeMed Medication Management System.

Figures1^4^ summarize the medication management procedures with SMMS. After each visit, the medications were collected by RCHE staff, who also entered residents’ information and medications into the database of the SMMS (Figure 1). A new profile was created for each resident in the SMMS. Their profiles were updated by RCHE staff after each follow-up at hospitals or GOPCs. During the medication preparation process, RCHE staff checked and verified the medications against the images shown on the electronic tablet (Figure 2). The medications are then administered to the residents after the RCHE staff verified the residents’ identifiers and medications on the electronic medication records on the tablet (Figure 3). When administering injectable medications, RCHE staff would check the previous injection site and record the current injection site on the tablet, as well as select the appropriate site for the next injection (Figure 4).

The SMMS aims to enhance the efficiency of and reduce potential medication errors in the medication management process (Table 1). It includes all medications commonly prescribed by clinicians from the HA. The system’s detailed drug database is regularly maintained by HKPCF pharmacists. The medication details available in the database include trade name, dosage form, strength, legal classification, and images of the medications. RCHE staff can verify the medications by comparing with the images provided by the system. Additionally, the system can generate administration schedules so that the medications can be delivered to residents in a real-time manner. It also supports electronic signatures for medication checking, preparation, and administration, eliminating the need for paper-based records.

**Table 1.** Features and advantages of the SMMS in the medication management workflow.

System features	Advantages
Data entry	
A comprehensive drug database with drug codes in the public health care system	Minimizes errors resulting from the manual data entry of drug names and doses.
Able to enter complex regimens and calculate the dispensing quantity automatically (eg, Warfarin 2.5 mg daily on odd days and 3 mg daily on even days)	Reduces errors and saves time by eliminating the need to manually calculate the dispensing quantity.
Automatically records the responsible personnel for each update in the resident profile and eMAR^a^ in the system	Enhances traceability and accountability.
Residents’ records	
A simple and readable format	Improves efficiency by reducing the time to search from paper MARs^b^.
Captures current medications from different prescribing sources	Quickly reviews resident’s past medication history. Improves efficiency and reduces errors by flagging up repeat prescriptions from residents (older adults with multimorbidity who consult multiple specialties from different specialty clinics [19]); thus, duplicate regimens may occur between different specialties even at the same health care institutions [19].
Real-time updating of the eMAR after any updates are made to the resident’s record	Improves the accuracy of checking and administration relative to the outdated paper MARs, which staff may need more time to check one by one or may even forget to update.
Medication preparation and checking	
A simple and readable format on tablets	Improves efficiency (reduces the time to search from paper MARs) and potentially reduces errors.
Provides images of medications and administration quantity	Improves efficiency and reduces errors as the RCHE^c^ staff can check the appearance and view the dispensing quantity of the medication immediately.
Real-time electronic signature	Improves accountability and saves time by eliminating the need to sign paper MARs one by one.
eMARs	
A simple and readable format on tablets	Improves efficiency (saves time by eliminating the need to search from paper MARs) and potentially reduces errors.
Real-time updates of medications to be administered to residents	Improves efficiency and reduces the chances of forgetting to update the paper MARs.
Shows only medications to be administered at a particular time during the time of drug administration	Improves efficiency as traditionally RCHE staff needed to search the paper MARs one by one to determine who needed to take medications in a particular time period. Helps to reduce errors in the timely administration of medications.
Real-time notification of late administration	Reduces errors in the timely administration of medications.
Provides photos of the residents and medications	Improves efficiency and reduces errors as the RCHE staff do not need to search the paper records one by one to identify the residents and medications.
Real-time electronic signature	Improves accountability and saves time by eliminating the need to sign the paper MARs one by one.
Group medications of different dosage forms under separate folders, including oral, nonoral, and injectable medications	Improves efficiency as each administration route may require different grades of nursing personnel to execute or supervise the administration.
Displays the last administration site and allows for selecting the current administration site for injectable medications and transdermal patches	Improves safety by facilitating administration site selection and rotation.

a eMAR: electronic medication administration record.

b MAR: medication administration record.

c RCHE: residential care home for the elderly.

### Outcomes

#### Time Efficiency

Time efficiency was evaluated using the time–motion analysis method, which has been used to analyze the efficiency of workflow in RCHEs [5,20]. A detailed description and sample size calculation for the time–motion analysis is presented in Table S1 in Multimedia Appendix 1.

To summarize, the investigators (HCC, PLWAD, and CHW) recorded videos of RCHE staff performing their usual duties in medication preparation, checking, and administration before and after the implementation of the SMMS. The recording protocol was developed based on recommendations from the literature [20] and had been published in our previous study [5]. A breakdown of the specific steps of the recording is presented in Table S2 in Multimedia Appendix 1.

#### Staff Competencies and Perceived Acceptance of the SMMS

The RCHE staff competencies in completing medication management procedures and their acceptance of the system were evaluated using a structured survey, which was developed based on the technology acceptance model, one of the most widely applied models to evaluate consumer acceptability of IT [21]. The model posits that perceived usefulness and perceived ease of use are important factors determining whether a newly introduced technology would be accepted by its potential users [21]. The questionnaire items were adapted from another local study [22] and reviewed by clinical pharmacists (SCC, HCC, and KCL) and survey methodologists (YTC and TTNL). A pilot study of the questionnaire was conducted with five RCHE staff, and the questionnaire was modified accordingly.

The competency part of the questionnaire comprised 4 domains: entering residents’ information, accessing residents’ records, preparing and checking medication, and administering medication. The RCHE staff rated their competencies in completing the tasks in the medication management process on a 10-point scale (−5=not able to complete at all, 0=same as traditional practice, and 5=able to complete). A higher score indicates a greater competency in completing the task. The competency survey was distributed to the RCHE staff a maximum of 12 weeks prior to the implementation of the program. RCHE staff was recruited through convenience sampling with the support of the RCHE management. The RCHE staff were asked to self-administer the survey. Each RCHE staff member was assigned a code to enable matching for the postintervention survey, while maintaining anonymity. Only the RCHE management has access to the identified matching list.

The acceptance part of the questionnaire comprised 4 domains: ease of use and technical support, perceived benefits to residents, perceived benefits to RCHE staff, and perceived support from RCHE management. The RCHE staff rated their acceptance on a 6-point scale (1=strongly disagree and 6=strongly agree). A higher score indicates a higher acceptance of the system. At the end, RCHE staff also provided an overall satisfaction score on a 10-point scale. The acceptance part of the questionnaire was only completed at the postintervention assessment.

The internal reliability of the scale was evaluated using Cronbach α and the item–total correlations based on 156 surveys conducted from September 2022 to February 2023. Cronbach α [23] and corrected item–total correlations [24] were calculated to measure the strength of the relationship between each item and the total score of the scale (Table S3 in Multimedia Appendix 1). The internal reliability for both surveys showed satisfactory psychometric properties.

#### Predictors of Staff Competencies and Perceived Acceptance

Data were collected on individual characteristics, namely, age, gender, working experiences, and qualifications [25], as well as organizational factors, namely, the financial model of operation and number of staff in each RCHE [16,26,27]. The organizational data were retrieved from the Hong Kong Government Social Welfare Department website [28].

#### Potential Cost Savings

An exploratory analysis was conducted to estimate the potential financial savings attributable to SMMS implementation using the official nursing staff salary data obtained from the Master Pay Scale of the Social Welfare Department’s (MPS) Salary Scale of common posts in nongovernmental organizations effective from April 1, 2024 [29]. The average monthly salary was calculated to be HKD 39,922.50 (US $5118) based on the midpoint salaries for registered nurses (MPS point 20; HKD 44,765 [US $5740]) and enrolled nurses (MPS point 15; HKD 35,080 [US $4498]). Assuming a standard working schedule of 176 hours per month, equivalent to a 44-hour work week, the average hourly wage was calculated to be HKD 227 (US $29), that is, approximately HKD 38 (US $4.9) per 10 minutes. The time–motion analysis provided the mean number of doses processed per 10 minutes before and after SMMS implementation and was used with the calculated salary per 10-minute period (HKD 38 [US $4.9]) to estimate the cost per dose prepared by RCHE staff. The difference in cost per dose before and after the system implementation was used to estimate potential labor cost savings associated with improved time efficiency in the medication management process.

### Statistical Analysis

The characteristics of the participating RCHEs and RCHE staff were summarized using descriptive statistics. The numbers of doses prepared, checked, and administered before and after the system implementation were stratified by implementation status and analyzed using a 3-way ANOVA. One sample 2-tailed *t* test (reference value=0, indicating no change) was used to compare the changes in staff’s competency before versus after the system implementation. The survey results were analyzed using the Mann-Whitney *U* test (categorical variables) and the Spearman correlation test (continuous variables) to identify individual and organizational factors associated with perceived competency and acceptance.

All analyses were performed using SPSS software, version 25. The results were considered statistically significant at *P* values of ≤.05.

## Results

### Baseline Characteristics of the RCHEs and RCHE Staff

Forty-one RCHEs participated in this study (Table 2). Nearly 90% (n=36) of the participating RCHEs were care and attention homes for older people. Twenty-five (61%) of them were subvented homes. Approximately 75% (n=31) of the participating RCHEs had more than 40 residents who were on at least one oral medication. The average RCHE nursing staff to resident ratio was 1:10. Regarding the baseline characteristics of the 3911 residents, 61.2% (n=2722) of them were female, their mean age was 83.8 years, and the median number of oral medications per resident was 11.

**Table 2.** Baseline characteristics of the participating RCHEs, residents, and RCHE staff.

Characteristics	Sample
RCHEs^a^ (n=41)	
Care level^b^, n (%)
Care and attention home for the older people	36 (87.8)
Others	5 (12.2)
Mode of operation, n (%)
Subvented home^c^	25 (61.0)
Self-financing home or private home	14 (34.1)
Contract home (with both subsidized and nonsubsidized places)	2 (4.9)
Number of residents in each RCHE with at least one oral medication, n (%)
1‐40 residents	10 (24.4)
41‐80 residents	9 (22.0)
≥81 residents	22 (53.6)
Average staff-to-resident ratio	1:2
Average nursing staff-to-resident ratio	1:10
Residents (n=3,911)	
Age of residents (y), mean (SD)	83.8 (12.3)
Gender, n (%)	
Male	1519 (38.8)
Female	2722 (61.2)
Oral medications per residents, median (IQR)	11 (8-15)
RCHE staff (n=392)	
Gender, n (%)	
Male	45 (11.5)
Female	278 (70.9)
Prefer not to answer	69 (17.6)
Age (y), n (%)	
19‐39 y	196 (50.0)
≥40 y	180 (45.9)
Prefer not to answer	16 (4.1)
Job nature, n (%)	
Full-time	329 (83.9)
Part-time	29 (7.4)
Mixed	34 (8.7)
RCHE staff working experience, n (%)	
<10 y	247 (63.0)
≥10 y	136 (34.7)
Missing	9 (2.3)
Working hours per day, median (IQR)	8 (5-10)
Qualification, n (%)	
Registered nurses or enrolled nurses	196 (50.0)
Health care workers or dispensers	175 (44.6)
Others	21 (5.4)
Duration between preimplementation assessment and SMMS implementation, median (IQR)	37 (4–57) days
Duration between SMMS implementation and postimplementation assessment, median (IQR)	253 (195–353) days

a RCHEs: residential care homes for the elderly.

b Care and attention homes for older people refer to RCHEs that provide personal care to older people with moderate impairment who are unable to live at home.

c Subvented homes refer to RCHEs that are operated by nongovernmental organizations and are subsidized by the government.

In the 41 participating RCHEs, 392 RCHE staff completed the pre–post surveys (response rate: 65%, loss-to-follow-up rate: 34%; Table 2). The median time between preintervention survey completion and system implementation was 37 days, while the median time for postintervention survey completion since system implementation was 253 days. Among the participants, 70.9% (n=278) were female. Half of the participants were between 19 and 39 years of age and were qualified as registered nurses or enrolled nurses. Additionally, 63% of the participants had less than 10 years of experience working in RCHEs.

### Time Efficiency

**Figure 5.**
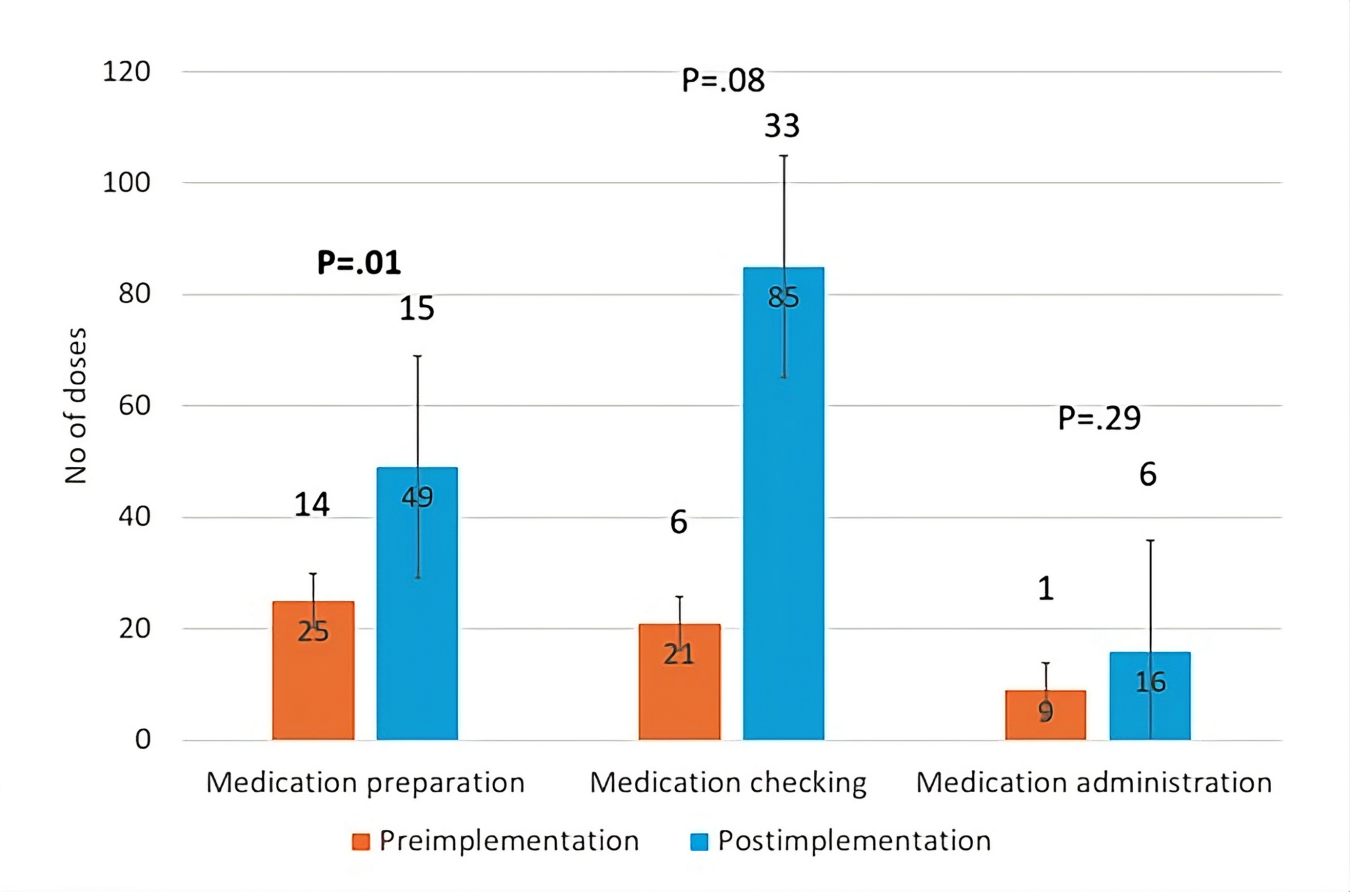
The ANOVA results revealed that the overall mean number of doses processed across all processes combined was significantly higher after the system implementation (18.9 vs 51.9 doses, *P*=.02). Additionally, the estimated time required (per 1000 doses) reduced for all processes after the system implementation: the time required for medication preparation decreased from 824.0 to 432.1 min; for medication checking, from 750.0 to 250.8 min; and for medication administration, from 1277.2 to 905.6 min.

Time–motion data from 770 minutes of recording were collected from six participating RCHEs. The average number of doses prepared, checked, and administered in 10-minute blocks all increased following the system implementation. Specifically, medication preparation increased significantly from 25 (SD 14) to 49 (SD 15) doses (*P*=.01; Figure 5). Medication checking increased marginally from 21 (SD 6) to 85 (SD 33) doses (*P*=.08), though the difference was not statistically significant. There was no significant change in the doses administered (9, SD 1 to 16, SD 6 doses; *P*=.29). The detailed breakdown of the time–motion analysis is presented in Table S4 in Multimedia Appendix 1.

### RCHE Staff Perceived Competency

Staff competencies in completing the medication management workflow improved significantly after the system implementation. This improvement was observed in the areas of entering and accessing residents’ records and in the processes of medication preparation, checking, and administration (all *P*’s<.001; Table S5 in Multimedia Appendix 1).

Staff competencies were not associated with gender, age, qualifications, work experience, or mode of operations (Table S5 in Multimedia Appendix 1).

### RCHE Staff Perceived Acceptance

The RCHE staff agreed that the system was easy to use and beneficial to the residents and them for their daily work (Table 3). The mean ratings were between 3.8 and 4.2 out of 6. The overall satisfaction score was 6.9 (SD 1.9) out of 10.

**Table 3.** RCHE staff perceived acceptance to the system. A Mann–Whitney test was conducted to compare staff-perceived acceptance by staff-level factors and institutional-level factors.

Domain	Ease of use and technical support^a^		Perceived benefits to residents^a^		Perceived benefits to RCHE^b^ staff^a^		Perceived support from RCHE management^a^		Overall satisfaction towards the system^a^	
	Mean (SD)	*P* value	Mean (SD)	*P* value	Mean (SD)	*P* value	Mean (SD)	*P* value	Mean (SD)	*P* value
Overall cohort (points)	3.8 (1.0)		4.2 (1.1)		3.9 (1.1)		4.1 (1.0)		6.9 (1.9)	
Comparison by staff-level factors:										
Qualifications		.85		.86		.54		.77		.02
Registered or enrolled nurses	3.8 (1.0)		4.2 (1.1)		4.0 (1.2)		4.1 (1.0)		6.7 (2.0)	
Health care workers or dispensers	3.9 (1.0)		4.2 (1.1)		4.0 (1.0)		4.2 (1.0)		7.2 (1.8)	
Gender		.24		.18		.29		.34		.02
Male	4.0 (1.1)		4.4 (1.0)		4.1 (1.4)		4.3 (1.2)		7.2 (2.1)	
Female	3.8 (1.1)		4.2 (1.1)		3.9 (1.1)		4.1 (1.0)		6.7 (1.9)	
Age (y)		.09		.22		.13		.29		.44
19‐39	3.9 (1.1)		4.3 (1.0)		4.1 (1.1)		4.1 (1.1)		6.8 (1.9)	
≥40	3.7 (0.9)		4.1 (1.2)		3.8 (1.1)		4.0 (1.0)		6.9 (2.0)	
RCHEs working experiences		.02		.69		.53		.23		.33
<10 y	3.9 (1.1)		4.2 (1.2)		4.0 (1.1)		4.2 (1.1)		6.9 (2.0)	
≥10 y	3.6 (0.9)		4.2 (1.0)		3.9 (1.1)		4.0 (1.0)		6.8 (1.8)	
Comparison by Institutional-level factors										
Mode of operation		.13		.44		<.001		.08		.25
Subvented home	3.9 (1.0)		4.2 (1.1)		4.1 (1.0)		4.2 (1.0)		6.8 (1.8)	
Self-financing home, private home, or contract home	3.6 (1.2)		4.1 (1.2)		3.4 (1.2)		3.8 (1.1)		7.0 (2.2)	
Number of residents who have at least one medication in each RCHE		.28		.63		.42		.36		.02
1‐40	3.8 (1.1)		3.9 (1.2)		3.8 (1.3)		3.8 (1.0)		7.3 (2.0)	
41‐80	3.7 (1.1)		4.2 (1.0)		3.8 (1.1)		4.1 (1.1)		6.5 (1.9)	
>80	3.9 (1.0)		4.2 (1.1)		4.0 (1.1)		4.1 (1.0)		6.9 (1.9)	

a The RCHE staff rated their acceptance on a 6-point scale (1=strongly disagree and 6=strongly agree) for all subscales, except for “overall satisfaction” on a 10-point scale. A higher score indicates a higher acceptance of the system.

b RCHE: residential care home for the elderly.

RCHE staff with ≥10 years of work experience gave a lower rating for the system’s ease of use than those with shorter experiences (3.6 [SD 0.9] vs 3.9 [1.1], *P*=.02). The staff from government-funded RCHEs tended to agree that the system benefited their daily work more than staff from RCHEs with other funding models (private, self-financing, or contract homes; 4.1 [1.0] vs 3.4 [1.2], *P*<.001). Staff in RCHEs with a smaller number of residents (1-40) were more satisfied with the system than those in larger RCHEs with >40 residents (7.3 [2.0] vs 6.5 [1.9] vs 6.9 [1.9], *P*=.022).

### Potential Cost Savings

The ANOVA results revealed significantly improved efficiency in the medication management process after SMMS implementation and that the mean doses processed per 10-minute interval increased from 18.9 (preimplementation) to 51.9 (postimplementation). Using a calculated labor cost of HKD 38 per 10-minute interval, the estimated cost per dose dropped substantially from HKD 2.00 (US $0.25) before to HKD 0.74 (US $0.09) after SMMS implementation.

The median number of oral medications per RCHE resident is 11. Assuming that each medication is taken, on average, twice daily, each resident requires 22 doses of medications prepared, checked, and administered daily. There are currently 58,500 residents in the RCHEs across Hong Kong [2]; hence, the system, if fully implemented in all RCHEs, could lead to a daily reduction in human resource costs associated with medication management from HKD 2,574,000 (US $330,000) to HKD 952,380 (US 122,100).

## Discussion

### Summary of Key Findings and Implications

This is the first and largest study in Hong Kong to evaluate the impact of a digital web-based system integrating electronic medical profiles and eMARs on time efficiency and potential human resource cost reduction from the perspectives of RCHE staff. Our results show that medications were more efficiently managed after system implementation across all processes combined, particularly during the medication preparation process, though improvement in medication checking and medication administration processes was marginal and statistically insignificant. The RCHE staff reported improved competency in completing the medication management process and supported the long-term use of the electronic system. The findings demonstrate that the system can address the current limitations in medication management processes in RCHEs.

The time–motion analysis findings show encouraging results as the number of packed and checked doses increased almost 3-fold from before to after the system implementation. This is a conservative estimate as this evaluation was conducted during the early implementation phase; the absolute efficiency would improve further once the RCHEs have had a longer run-in and usage time. Efficiency was improved because the paperless processes had replaced the paper-based processes to eliminate the need for physical signatures on each paper MAR during medication packing and checking. eMARs display only the medications that need preparation at a specific time, significantly reducing the time required to verify the medications (Table S4 in Multimedia Appendix 1). Furthermore, structural previews and automatic calculations for dispensing quantities contributed to increased time efficiency, particularly for complex medication regimens. Notably, the SMMS appeared to have a less significant impact on medication administration, as the considerable variation in this process may be influenced by environmental and patient factors. For example, RCHE staff might still prefer to perform manual and visual inspections of medication and patient identifiers as a safety measure, even though these processes are supported by the features of the SMMS. Nevertheless, the RCHE staff felt satisfied with the system. This demonstrates that the integration of IT into daily routines can enhance the efficiency of the medication management process, allowing staff to contribute more time to frontline nursing care.

The exploratory analysis showed that the professional labor costs associated with medication handling in RCHEs may potentially lead to a daily reduction of HKD 1,621,620 (US $207,900) in human resource costs associated with medication management in RCHEs. We acknowledge that this estimate should be interpreted cautiously, given the assumptions underlying this analysis. These assumptions include the scaling of the program with consistent workflow to all RCHEs in Hong Kong, as well as considerations related to the costs of purchasing, installing, and maintaining the system, along with training expenses, among other factors. However, this exploratory analysis suggests that such financial benefits could allow residential care facilities to redirect available resources toward other critical areas, including enhancing staff training, improving patient care quality, and addressing urgent staffing needs. This is especially encouraging as there is currently a shortage of health care personnel in the primary care setting of Hong Kong. This system is in line with the recently released Hong Kong Primary Healthcare Blueprint [30], which explicitly acknowledges the need for innovative technologies, including automation and artificial intelligence, to alleviate workforce burdens. The SMMS has the potential to directly reduce the workloads of nursing staff and spare their human resources for other duties.

Our results reveal that the RCHE staff reported significantly improved competencies in performing different aspects of the medication management process using the SMMS. Our findings align with recent studies showing that a considerable proportion of RCHE staff hold positive perceptions of the use of electronic systems [31], though this result should be interpreted cautiously, given that the competency assessment tool has not been extensively validated. The time between competency survey completion and system implementation varied widely among the RCHE staff, which may have resulted in inaccuracies and recall bias. However, the competency findings are still important in shaping future optimization of the SMMS as the tool examines staff competency in handling specific features of the intervention based on the widely used technology acceptance model. For example, the RCHE staff acknowledged that the system offers substantial benefits for residents by enhancing the safety of the medication management process through the automatic calculation of dispensing quantity and detection of duplicate prescriptions and late administration. Future work should conduct in-depth studies on the user experience using conceptual implementation science framework specific to technology adoption, such as the Unified Theory of Acceptance and Use of Technology and the Consolidated Framework for Implementation Research to assess the barriers and facilitators of the SMMS implementation success [32,33].

We also acknowledge that the current evaluation did not include safety and reduction in medication errors as an outcome due to the difficulty in capturing self-reported errors and “near-misses” in a large-scale and consistent manner during the preimplementation phase. However, our previous pilot study demonstrated a significant reduction in medication errors (preimplementation: 10/9504 doses, 0.1%; postimplementation: 0/5731 doses; *P*=.02) [5]. The study also found that the SMMS effectively identified one-third of the residents as taking potentially inappropriate medications based on the Beers criteria [34]. Future work should focus on quantifying the medication safety aspects of the SMMS, particularly in facilitating pharmacists-led medication reviews, establishing referral pathways to promote deprescribing and therapy modifications, and computerized decision-support tools to reduce potentially inappropriate prescriptions and the use of potentially inappropriate medications in this special population.

The literature presents varying findings regarding the factors associated with RCHE staff’s acceptance of new technology. For example, some studies have shown that female or older RCHE staff and those with longer work experience might have lower computer literacy and a lower acceptance of new technology [35]. Our study did not identify any significant association between staff demographic characteristics and competency, suggesting that most of the RCHE staff considered the system user-friendly and manageable. However, we found that institution-level factors were associated with staff acceptance of and satisfaction with the system. Importantly, the RCHE staff from self-financing and private RCHEs were less likely than the RCHE staff from subvented homes to agree that the SMMS was beneficial for their daily work. This could be due to a higher expectation of quality and less subsidized support in private RCHEs than in government-funded RCHEs. Small-scale RCHEs (with ≤40 residents) had a higher satisfaction with the system than large-scale RCHEs, probably due to a less demanding workload. Our results indicate a strong association between management support and staff satisfaction with the system, suggesting that effective communication between the management team and frontline staff can facilitate this transition in the early implementation phase. More in-depth interviews with RCHE staff and stakeholders from different operation modes are needed to explore their expectations of and support for the system.

### Limitations

This study has several limitations. First, only 6 RCHEs participated in the time–motion study. One reason may be the ongoing COVID-19 pandemic and the infection control measures implemented during the preimplementation phase, which affected the willingness of RCHEs to participate in the time–motion analysis. Nevertheless, this is still considered a reasonably adequate sample size as few studies in the literature have systematically evaluated the effect of automation on time efficiency at an institutional level. Furthermore, as the six participating RCHEs are all subsidized facilities, the findings may be generalizable to other government-subsidized homes, which represent the majority of funding models for RCHEs in Hong Kong. Second, the Hawthorne effect may have influenced the participants’ behavior while under observation, potentially leading to the recorded number of doses not accurately reflecting the true situation [36]. Therefore, attempts were made to record more than one staff per nursing home, and the videos were limited to less than 30 minutes per staff to reduce their stress levels. The improvement in outcomes may be a “registration effect,” suggesting that the initial improvements could diminish over time. However, considering that the average run-in period between implementation and evaluation was 6 to 12 months, the findings may reflect the program’s effects in the intermediate term. A longer follow-up study is needed to assess the sustainability of the program’s outcomes.

The potential for sampling bias cannot be ruled out as the participants who completed the survey may have had a greater awareness of the new system implementation than those who did not complete the survey. Although a proportion of the RCHE staff might have missed the survey due to conflicting schedules, we could not determine the reasons for the unwillingness to respond among RCHE staff who explicitly declined to participate in the survey. However, a moderate response rate (65%) is acceptable in most survey studies involving convenience samples. Lastly, unlike our pilot study [5], we did not capture the rate of medication errors, as this exercise likely led to underreporting and a reluctance among RCHE staff to report errors or near-misses. However, it is reasonable to postulate that the rate of medication errors could be effectively minimized due to the additional layers of safety nets outlined in Table 1.

### Future Work

To further explore how the digital medication management system could reduce costs compared with traditional workflows, our next step involves investigating the actual cost savings achieved through reduced dispensing and preparation work in the medication management process. This work also lays the foundation for the potential integration of electronic health and medication records in the public health care system with the SMMS in RCHEs. This integration could enable a seamless, closed-loop medication management system that encompasses prescribing, dispensing, preparing, and administering. Future work could extend the application of the system to other settings that involve complex medication management, such as rehabilitation centers and long-term care facilities for individuals with special needs.

In 2020, the Hong Kong government released “The Smart City Blueprint for Hong Kong 2.0” to expand IT applications to enhance the quality of life of older people [37]. A notable initiative highlighted within the Blueprint is the establishment of the HKD 1 billion Innovation and Technology Fund for Application in Elderly and Rehabilitation Care (I&T Fund), initiated in 2018 [38]. This fund aims to encourage older people care units to adopt recognized gerontechnology solutions, including digital tools that streamline medication management and electronic health records to reduce the burden and pressure on care staff. The SMMS was officially listed as a “Recognized Technology Application Product” in the Social Welfare Department’s technology directory, under the category of computerized medication management systems [39]. This government-level recognition indicates government stakeholders’ acknowledgment of the potential of the SMMS to enhance the quality of older people care through reducing medication errors and streamlining operational workflows. Our findings provide policymakers with insights to allocate additional funding for implementing IT in RCHE medication management. These results support expanding and integrating “smart living” technologies into various operation modes of RCHEs. Future efforts should focus on collaboration with policymakers to address potential barriers such as hardware or resource supports for the system implementation.

### Conclusions

This is the first study in Hong Kong to evaluate the impact of using a digital web-based system integrating eMARs from the perspectives of RCHE staff. Our results show that the system was effective in improving time efficiency and significantly enhancing competencies in medication preparation, checking, and administration. This highlights the importance of implementing electronic systems in RCHEs, guiding relevant stakeholders and policymakers tasked with enhancing the safety and efficiency of medication management for the aging population.

## Supplementary material

10.2196/79262 Multimedia Appendix 1 Additional information on methodology and results.

## Abbreviations

eMAR: electronic medication administration record
GOPC: general outpatient clinics
HA: Hospital Authority
HKPCF: Hong Kong Pharmaceutical Care Foundation Ltd
MPS: Master Pay Scale of the Social Welfare Department
RCHE: resident care homes for the elderly
SMMS: SafeMed Medication Management System
