# Supplementary material for: Implementation of an Electronic Medication Management System in 41 Residential Care Homes in Hong Kong: Pre–Post Interventional Study
Source: JMIR Aging. 2025 Dec 4;8:e79262. doi: 10.2196/79262 (PMC12677979; doi:10.2196/79262)
Supplement: Multimedia Appendix 1 [file aging-v8-e79262-s001.docx]

**Table S1: Procedure for Time-motion Analysis and Ethical Considerations**

As it was not logistically feasible to conduct time -motion analysis on all RCHEs, a sample size calculation was conducted based on our previous pilot study[5], which considered the expected effect size (mean change in doses prepared and checked combined = 53.3; moderate effect size = 0.7), the variability in the time taken for medication management tasks (SD = 11.4), and 80% as desired power of the study. Using a two-tailed t-test with an alpha level of 0.05, a minimum of 8 observations would be required to detect a meaningful difference in time efficiency before and after the implementation of the SMMS®. It was decided a priori that the RCHEs would be invited to participate in the time motion analysis through consecutive sampling until the desired number was reached. However, this was not feasible at the start of the program due to the COVID pandemic, which resulted in infection control measures that restricted research personnel from entering RCHEs from January 2022 to March 2023. Since April 2023, a total of 19 invitations were sent, but 7 RCHEs declined to consent to video recording and 6 could not complete pre-intervention recordings due to limited run-in time and other logistical barriers. Finally, 6 RCHEs agreed to participate and were included in the time motion analysis.

We obtained verbal consent of staff members observed in old age homes (OAHs) and the Hong Kong Pharmaceutical Care Foundation (HKPCF) was obtained prior to each data collection period. We adopted verbal consent to ensure that all data were obtained anonymously. The following points were addressed in verbal consent:

|  | Points addressed in the verbal consent |
| --- | --- |
| 1 | Participation was voluntary and implied consent to the terms and conditions of the study. |
| 2 | Participation involved being observed and videotaped the usual work on medication preparation, checking and administration. No interruptions were made during working. |
| 3 | All data were collected anonymously. No facial appearance was videotaped during time-motion observations. |
| 4 | No punishment was incurred due to errors observed and reported in the study. |
| 5 | Participants had the right of withdrawal from the study at any time. |
| 6 | All data collected were only used for research purposes and were not publicly disclosed in a fashion that would identify any specific person or organization. |
| 7 | The researcher team took precautions to preserve the confidentiality of the research data and that all reports of the research would be devoid of identifiers. |

As one of the terms and conditions in the service agreement, RCHEs receiving the service had agreed the collection and use of data available in SafeMed Medication Management System (SMMS®), including residents’ **anonymous** medication records and data for medication wastage, prior to the implementation of the Integrated Old Age Home Medication Management Programme.

Our pilot study did not record videos during the administration process because of resident confidentiality concerns [5]. To address this limitation, investigators obtained approval from the RCHE superintendents and ensured that no resident identities or faces were captured in the recordings. We obtained verbal consent of staff members observed in RCHEs prior to each data collection, with the above key ethical considerations emphasized for each consent-taking process. All data were collected anonymously. No facial appearance was videotaped during time-motion observations. Efforts were deliberately made to ensure that the videos focused solely on the hands performing the tasks, rather than on the faces. The data collection post-SMMS implementation was conducted at least 2 weeks after the system implementation. The videos were analyzed in 10-minute blocks, and the mean number of doses prepared, checked, and administered were calculated for each block.

**Table S2: Recording Protocol for Time Motion Analysis**

| **1)     Preparation of medication** | |
| --- | --- |
| Preparation of medications refers to the transferring oral solid medications from any bulk storage (e.g. resident’s original medicine bags) to a container readily available for administration at the home) | |
| *Pre-Programme* | |
|  | Setting up area, equipment and documentation for preparation of medications |
|  | Take residents’ solid oral medications from residents’ storage bins/drawers |
|  | Allocate residents’ medication to suitable container |
|  | Documentation of medication preparation on paper medication records |
| *Post-Programme* | |
|  | Setting up area, equipment |
|  | Loading digital records on electronic medication records on tablets |
|  | Take residents’ solid oral medications from residents’ storage bins/drawers |
|  | Allocate residents’ medication to suitable container |
|  | Documentation of medication preparation on electronic medication records with digital signature |
| **2)     Checking of prepared medication** | |
| Checking of prepared medication, which happens right after preparation and some time (e.g. a day in advance) before administration | |
| *Pre-Programme* | |
|  | Setting up area, equipment and documentation for checking of prepared medications |
|  | Checking of prepared medications |
|  | Correction of wrongly prepared/changed medications (if any) |
|  | Storage of prepared medications |
|  | Documentation of medication checking using wet ink signature |
| *Post-Programme* | |
|  | Reception and storage of finished product in container |
|  | Setting up area, equipment and documentation for checking of prepared medications |
|  | Checking of prepared medications against images on tablets |
|  | Correction of wrongly prepared/changed medications (if any) |
|  | Storage and sorting of prepared medications |
|  | Documentation of medication checking using digital signature |
| **3)     Administration of medications** | |
| Administration of any medications, including prepared or unprepared, of any dosage forms, to the residents | |
| *Pre-Programme* | |
|  | Preparing paper medication records, prepared medications and equipment for administration |
|  | Acquiring non-solid oral medications, checking site of injection on paper records |
|  | Acquiring prn medications |
|  | Crushing of medications |
|  | Verifying resident profile using paper records and physical labels |
|  | Administering medications to resident |
|  | Observing resident after administration |
|  | Documentation of administration using wet ink signature |
| *Post-Programme* | |
|  | Loading profile on electronic tablet, prepared medications and equipment for administration |
|  | Acquiring non-solid oral medications, checking site of injection on electronic tablet |
|  | Acquiring prn medications |
|  | Crushing of medications |
|  | Verifying resident profile using electronic tablet |
|  | Administering medications to resident |
|  | Observing resident after administration |
|  | Documentation of administration using digital signature |
| **4)     Others** | |
|  | Transit |
|  | Movement from one place to another while none of the above activities are being carried out |
|  | Interruptions |
|  | Any demands that cause the staff to deviate from the above activities (e.g. telephone calls, questions from other staff, resident emergencies) |
|  | Uncategorized |
|  | Any other activities that are deemed necessary to be recorded but cannot be categorized by any activity codes above (please provide a remark) |

**Table S3: Internal reliability of the perceived competency and acceptance survey**

The internal reliability of the scale was evaluated using Cronbach’s alpha and the item-total correlations based on 156 surveys conducted from September 2022 to February 2023. Cronbach’s alpha values of > 0.70 are considered satisfactory [24]. Corrected item–total correlations were calculated to measure the strength of the relationship between each item and the total score of the scale. A value of > 0.3 indicates an acceptable correlation with the total score of the scale [25]. The Cronbach’s alpha values for most of the domains were > 0.70 (range = 0.89–0.97), except for one subscale that had a Cronbach’s alpha of 0.625. The correlations were moderate or high (r > 0.5) for all of the items.

| **Items** | **Cronbach’s alpha** | **Corrected Item-Total Correlation** |
| --- | --- | --- |
| **Competency scale** | | |
| **Domain 1: Entering residents’ medication records** | 0.928 |  |
| 1.1 Efficiently input medication information from each follow-up prescription |  | 0.609 |
| 1.2 Accurately input medication information from each follow-up prescription |  | 0.828 |
| 1.3 Record complex prescriptions |  | 0.810 |
| 1.4 Accurately record medication duration and administration dates |  | 0.857 |
| 1.5 Accurately record medication sources (e.g., general outpatient clinic, specialist clinic) and prescription dates |  | 0.797 |
| 1.6 Accurately record the site of administration for topical medications |  | 0.793 |
| 1.7 Accurately record medication precautions |  | 0.836 |
| 1.8 Clearly record the individuals accountable for each medication management procedure |  | 0.781 |
| **Domain 2: Accessing residents’ records** | 0.896 |  |
| 2.1 Efficiently review the patient's past medication history |  | 0.760 |
| 2.2 Accurately identify any changes to the patient's medication |  | 0.773 |
| 2.3 Accurately identify duplicate medications |  | 0.696 |
| 2.4 Check for drug interactions (If there are "interacting medications" occurring simultaneously, the system will show the reason for the "interaction automatically) |  | 0.806 |
| 2.5 Efficiently access dispensing, checking, and administration records |  | 0.712 |
| **Domain 3: Preparing and checking medications** | 0.971 |  |
| 3.1 Accurately perform the dispensing process according to the prescribed time of administration |  | 0.785 |
| 3.2 Ensure the correct medication is dispensed |  | 0.882 |
| 3.3 Ensure the correct quantity of medication is dispensed |  | 0.912 |
| 3.4 Efficiently perform the three-check-five-rights process |  | 0.907 |
| 3.5 Accurately perform the three-check-five-rights process |  | 0.921 |
| 3.6 Accurately sign the records after dispensing for each resident |  | 0.927 |
| 3.7 Accurately sign the records after checking for each resident |  | 0.928 |
| **Domain 4: Administering medications** | 0.973 |  |
| 4.1 Efficiently administer medications |  | 0.920 |
| 4.2 Accurately perform the “five rights” (resident’s name, medication name and form, dosage, administration time, and route) |  | 0.928 |
| 4.3 Correctly administer PRN (as-needed) medications |  | 0.886 |
| 4.4 Always know whether any medication is missed |  | 0.784 |
| 4.5 Clearly aware of the sites of administration for topical medications |  | 0.850 |
| 4.6 Clearly record the application sites of patches and  Insulin |  | 0.852 |
| 4.7 Clearly record conditional instructions (e.g, blood pressure, heart rate) |  | 0.909 |
| 4.8 Clearly understand precautions of medication |  | 0.922 |
| 4.9 Accurately sign the records after administering medication for each resident |  | 0.945 |
| **Perception of acceptance to SMMS®** | | |
| **Domain 1: Ease of use and technical support** | 0.887 |  |
| 1.1 I can easily understand how to operate the new system |  | 0.815 |
| 1.2 HKPCF provides sufficient training |  | 0.798 |
| 1.3 I clearly know how to troubleshoot the issues I encounter in the system. |  | 0.819 |
| 1.4 My nursing home has sufficient hardware to support the system (e.g. Wi-Fi, computer systems) |  | 0.597 |
| **Domain 2: Perceived benefits to residents** | 0.942 |  |
| 2.1 The system can minimize mistakes resulting from inputting medication. |  | 0.845 |
| 2.2 The system can reduce dispensing and packaging errors |  | 0.915 |
| 2.3 The system can reduce medication administration errors |  | 0.879 |
| **Domain 3: Perceived benefits to RCHE staff daily work** | 0.910 |  |
| 3.1 The system can help me complete medication management tasks |  | 0.846 |
| 3.2 The system can simplify medication management, giving me more time to other care duties for residents |  | 0.805 |
| 3.3 The system is able to present the patients' medical records and past medication data in a clear and concise format |  | 0.823 |
| **Domain 4: Perceived support from RCHE management** | 0.625 |  |
| 4.1 Most of my colleagues welcome the system |  | 0.457 |
| 4.2 The management of my nursing home supports this project |  | 0.457 |

RCHE: residential care home for the elderly; SMMS®: SafeMed Medication Management System®

**Table S4: Breakdown of the time-motion analysis results during medication preparation, checking and administration**

| **Pre implementation steps** | **Mean [SD] duration per medication (sec)** | **Post implementation steps** | **Mean [SD] duration per medication (sec)** |
| --- | --- | --- | --- |
| ***Medication preparation*** | | |  |
| Transferring medications from original packaging into medication cups and verifying prepared medication according to paper medication records | 10.2±20.6 | Transferring medications from original packaging into medication cups and verifying prepared medication against medication name according to electronic medication record on tablet | 4.5±3.4 |
| Verification of resident’s profile on paper medication records | 7.4±6.7 | Verification of resident’s profile on electronic medication record on tablet, loading electronic profile | 4.6±7.7 |
| ***Medication checking*** | | |  |
| Checking prepared medication against labels on original packaging | 15.6±17.0 | Checking prepared medication against images of medications on tablet | 3.1±5.9 |
| Documentation by ink on paper medication records, wet ink signature | 2.3±3.4 | Documentation on electronic medication record, digital signature | 1.6±3.1 |
| ***Medication administration*** | | |  |
| Identifying individual resident via paper resident record and physical labels | 9.2±16.4 | Identifying individual resident via medication administration record on tablet | 1.7±1.9 |
| Checking current medication (type and quantity) on paper medication records | 19.5±12.5 | Checking current medication (type and quantity) on medication administration record on tablet | 12.8±11.7 |
| Administering each medication in the appropriate dosage form to the resident and signing the medication administration record using wet ink signature | 16.0±20.8 | Administering each medication in the appropriate dosage form to the resident and signing the medication administration record using digital signature | 7.3±11.5 |

**Table S5: RCHE staff competencies toward medication management workflow after system implementation**

| Competency Domain | Entering residents’ medication records* | | Accessing residents’ records* | | Preparing and checking medications* | | Administering medications* | |
| --- | --- | --- | --- | --- | --- | --- | --- | --- |
| Overall cohort: | mean ± SD | *P ^a^* | mean ± SD | *P ^a^* | mean ± SD | *P ^a^* | mean ± SD | *P ^a^* |
| Total score (points) | 3.5 ± 1.3 | **<.001** | 3.4 ± 1.4 | **< .001** | 3.8 ± 1.3 | **.001** | 3.8 ± 1.2 | **.001** |
| Comparison by staff-level factors: | mean ± SD | *P* ^b^ | mean ± SD | *P* ^b^ | mean ± SD | *P* ^b^ | mean ± SD | *P* ^b^ |
| Qualifications |  | .45 |  | .25 |  | .58 |  | .35 |
| Registered or enrolled nurses | 3.5 ± 1.3 |  | 3.3±1.4 |  | 3.7±1.3 |  | 3.8±1.1 |  |
| Health care workers or dispensers | 3.6 ± 1.3 |  | 3.5±1.4 |  | 3.8±1.4 |  | 3.8±1.4 |  |
| Gender |  | .27 |  | .43 |  | .90 |  | .93 |
| Male | 3.1 ± 1.7 |  | 3.1±1.8 |  | 3.4±2.0 |  | 3.6±1.7 |  |
| Female | 3.5 ± 1.2 |  | 3.4±1.3 |  | 3.8±1.2 |  | 3.8±1.1 |  |
| Age (years) |  | .35 |  | .47 |  | .69 |  | .43 |
| 19 – 39 | 3.5 ± 1.2 |  | 3.4 ± 1.3 |  | 3.8 ± 1.2 |  | 3.8 ± 1.1 |  |
| ≥ 40 | 3.5 ± 1.5 |  | 3.4 ± 1.5 |  | 3.7 ± 1.4 |  | 3.8 ± 1.4 |  |
| Working experiences in RCHEs |  | .67 |  | .78 |  | .43 |  | .67 |
| Less than 10 years | 3.5 ± 1.2 |  | 3.4 ± 1.3 |  | 3.8 ± 1.2 |  | 3.8 ± 1.2 |  |
| ≥10 years | 3.5 ± 1.4 |  | 3.4 ± 1.6 |  | 3.8 ± 1.5 |  | 3.8 ± 1.4 |  |
| Comparison by institutional-level factors: | mean ± SD | *P* ^b^ | mean ± SD | *P* ^b^ | mean ± SD | *P* ^b^ | mean ± SD | *P* ^b^ |
| Mode of operation |  | .72 |  | .40 |  | .11 |  | .52 |
| Subvented Home | 3.5 ± 1.3 |  | 3.4 ± 1.4 |  | 3.7±1.4 |  | 3.8±1.3 |  |
| Self-financing Home, Private Home or Contract Home | 3.4 ± 1.3 |  | 3.3 ± 1.3 |  | 3.9±1.2 |  | 3.8±1.2 |  |
| Number of residents who have at least one medication in each RCHE |  | .12 |  | .52 |  | .88 |  | .48 |
| 1 – 40 | 3.2 ± 1.8 |  | 3.3 ± 1.8 |  | 3.5 ± 1.9 |  | 3.5 ± 1.9 |  |
| 41 – 80 | 3.3 ± 1.4 |  | 3.3 ± 1.4 |  | 3.7 ± 1.3 |  | 3.7 ± 1.2 |  |
| > 80 | 3.6 ± 1.1 |  | 3.5 ± 1.3 |  | 3.8 ± 1.1 |  | 3.9 ± 1.0 |  |

RCHE: residential care home for the elderly. Subvented homes refer to RCHEs that are operated by NGOs and are subsidized by the government.

*^a^* The RCHE staff rated their competencies in completing the tasks in the medication management process on a 10-point scale (-5 = not able to complete at all, 0 = same as traditional practice, 5 = able to complete). A higher score indicates a greater competency in completing the task. One sample t-test (reference value = 0, indicating no change) was used to compare the changes in staff’s competency before versus after the system implementation.

^b^ Mann–Whitney test was conducted to compare staff perceived competency by staff-level factors and institutional-level factors.
